# 

**Published:** 1912-05-11

**Authors:** 


### OUR BUREAU OF INFORMATION.

### Cubic Air Space in Government Hospitals.

Mr. John B. Prest, Office Superintendent, State Board of Charities, New York, writes: I have been reading in The Hospital of March 30 on page 663 of the overcrowding in Poor-Law infirmaries, and note under the heading "The General Moral" the following sentence: "Certain alterations to windows and so forth have to be made, together with the reduction of the present number of beds, which, though more or less allowable when their occupiers are healthy, do not provide sufficient cubic space of air to provide for the Government regulations for the sick." This leads me to inquire what are the Government regulations for the sick in regard to cubic space of air, and I take the liberty to ask if you can send me a copy of these regulations, or forward this letter to the proper official who will send me a copy? The question of proper cubic space of air in hospital wards has been under consideration in New York for a year or more, and not long ago the State Board of Charities of New York formulated rules whereby the wards in hospitals caring for public patients would be licensed for a certain number of beds, the number being based upon the cubical space of the room.

The point on which information is desired is one of considerable public interest. No Government regulations have been framed in this country to which all institutions for the sick are bound to conform. It is open to any person to maintain either by private means, public subscriptions, or payment from patients any kind of establishment for the sick in which any kind of treatment may be administered to any description of adult sane patient, under any conditions which the proprietor or manager thinks fit. The only limit to this liberty of the subject is that imposed by the free will of those who enter such establishments. Under the Public Health (London) Act, 1891, the London County Council are able to deal summarily with any premises in such a state as to be a nuisance or dangerous to health, but it appears to be uncertain whether this provision would apply in the case of hospitals. The voluntary hospitals are, therefore, entirely unfettered in regard to the air space they provide for their patients.

Hospitals directly or indirectly under Government control fall under two headings: those provided by the War Office and those established under the jurisdiction of the Local Government Board, including those directed under the Metropolitan Asylums Board in London and other municipal authorities outside London.

The regulations of the War Office in regard to the space to be allotted to patients in military hospitals at home stations are laid down in paragraph 180 of the Regulations for the Army Medical Service, 1906, as follows:

| | Floor Space. Square feet. | Cubic Space. Cubic feet. |
|---|---|---|
| For permanent hospitals, light case wards ... ... | 65 | 900 |
| For permanent hospitals, ordinary wards ... ... | 85 | 1,200 |
| For permanent hospitals, infectious wards ... ... | 144 | 2,000 |
| For detached wooden huts, all wards ... ... ... | 70 | 800 |

At stations abroad a special scale is authorised for each command.

It is worth noting that the Local Government Board relies on public opinion to a large extent in dealing with the multiplicity of public bodies with which it is in relation, and couches its directions in the form of "Memoranda" or "Suggestions" or "Points to be Attended to" rather than as authoritative regulations. The result of this very English mode of dealing with public problems is undoubtedly to encourage initiative in municipalities and local bodies. In places where the right kind of guardian or town councillor influences public opinion, valuable experiments are made and the public hospitals reach a high pitch of excellence. It is impossible to deny, on the other hand, that a large number—perhaps the majority—of local bodies make a practice of sailing as near the wind as the vigilance of the Local Government Board inspectors will allow.

The "Suggestions" put forth by the Board in respect to air space are identical for Poor-Law infirmaries and for sick wards attached to workhouses. They were issued in 1891 in the form of a memorandum entitled "Points to be Attended to in the Construction of Workhouse Buildings." The passage respecting air space is as follows:

| | Wall Space.* Linear feet. | Floor Space. Square feet. | Cubic Space. Cubic feet. |
|---|---|---|---|
| Wards for ordinary sick ... ... | 6 | 60 | 600 |
| Itch and venereal ... ... ... | 6 | 60 | 600 |
| Wards for lying-in ... ... ... | 8 | 80 | 960 |
| Offensive cases ... ... ... | 8 | 80 | 960 |
| Children ... ... ... ... | 5 | (if the wards are 20 feet in width) | |
| Children ... ... ... ... | 6 | (if the wards are 18 feet in width) | |
| Isolation wards ... ... ... | 12 | 144 | 2,000 |

* Irrespective of doors and fireplaces.

In regard to isolation hospitals provided by means of loans sanctioned by the Local Government Board a more authoritative tone is taken. A memorandum,† issued in 1902 "On the Provision of Isolation Hospital Accommodation by Local Authorities," lays down very definite conditions which must be complied with before the Local Government Board will sanction loans.

"In the ward blocks each bed must have at least 12 linear feet of wall space, 144 square feet of floor space, and 2,000 cubic feet of air space. In calculating the latter any height of wards above 13 feet should not be taken into account."

An appendix was issued to this memorandum in 1908, which gives a plan for an observation block intended to provide isolation accommodation for single cases of infectious disease, for cases of mixed infection, or for cases of doubtful diagnosis. It is to be noted that this plan only provides 1,440 cubic feet of air space per bed instead of the 2,000 cubic feet required in ward blocks. This has been permitted in view of the separation of individual patients and the efficient cross-ventilation which the plan provides.

†To be obtained from Messrs. Eyre and Spottiswoode, East Harding Street, Fleet Street, London, E.C.

### NEEDS AND HELPERS.

Pensions and Sick Allowances.

Miss Ross, Inverness, should write to Mr. Louis Dick, 15 and 16 Buckingham Street, Strand, London, W.C., and he will send her the fullest particulars relating to the Royal National Pension Fund for Nurses, and give her every facility for introducing her staff to its benefits. An approved benefit society is being formed in connection with the Fund to deal with insurance.

Hip and Spinal Disease Cases.

Mr. G. H. Hodges, Cardiff, inquires "what hospitals or homes there are available at a low charge for cases of hip and spinal disease, requiring long stay without surgical treatment, when the patients are over twelve years of age. There are several places where children under this age are admitted at 4s. or 5s. a week." Have you tried the Hospital and Home for Incurable Children, Northcourt, Hampstead? The age there is under sixteen, and patients are admitted for "a small weekly payment." It is under excellent management and has fifty-six beds. Perhaps one of our readers can suggest a home nearer your locality. We do not know of any answering to your wants in Wales.

Inspection.

"D. B.," Maidstone.—We have arranged for a visit to be paid to you, but are sorry we cannot mention the day when our correspondent will be able to call.

Maitland Sanatorium.—Many thanks for kind letter. We fear that all the sanatoria are in the position you describe of having so large a number of ex-patients on their books that there are no openings for others who desire to lead the life and can pay a small sum, giving services in return.

Homes Wanted at Moderate Terms.

"C. F. S."—For a lady who has been treated at Duxhurst, but has now overcome the tendency to alcoholism [103a].

"Francis."—For a girl of 14, who has developed, somewhat suddenly, a bad type of neurasthenia, taking the form of religious delusions and melancholia. Fairly strong in health, affectionate, very conscientious, and anxious to overcome morbid feelings. South of England desirable though not essential [104a].

"F. L.," Forest Gate.—A temporary home for a baby, the mother of whom is unable to retain it with her, owing to the nature of her present occupation. Reasonable payment would be made [105a].

Replies to the above from Approved Homes should state terms and full particulars in a letter addressed to the initials or pseudonym given above and enclosed in a stamped envelope, together with card or slip of paper containing name and address for identification, to the Editor, The Hospital Bureau of Information, 28 Southampton Street, Strand, London. Proprietors of homes which have not yet been entered on our List of Approved Homes, but have accommodation to offer likely to suit any of the above applicants, are invited to write for an application form, that their replies may be forwarded as soon as references have been taken up. Correspondents will readily understand that we cannot assume the responsibility of putting them into communication with homes without first verifying the *bona fides* of those who offer accommodation.

"Mrs. D."—Can any of our readers offer a home for a small payment to a lady, aged 32, willing to undertake light secretarial or other duties in addition? Her lungs have been affected, but she has made a good recovery. She has had considerable experience in social work, and is efficient and conscientious in all she undertakes. Highest references. Prepaid replies, addressed under cover to the Editor of The Hospital Bureau of Information, forwarded.

### Appointments.

Dr. R. W. Richards, M.D., has been appointed medical officer for the Llandudno district. Dr. Richards is an M.D. of London University, and can also speak Welsh.

Dr. Frank Edward Tylecote, M.D., D.P.H. (Vict.), M.R.C.P. (Lond.), has been appointed second visiting physician to the South Manchester Workhouse Infirmary, Withington.

Dr. Cuthbert Balfour Paul, assistant surgeon to the Royal Hospital for Sick Children, Edinburgh, has been appointed honorary assistant surgeon to the Cumberland Infirmary.

The following appointments at the Manchester Royal Infirmary have been confirmed: Dr. George Ashton, M.D. (Vict.), re-appointed medical officer for home patients, and Mr. J. C. Jefferson, M.R.C.S., L.R.C.P., appointed junior house surgeon.

